# Nondestructive in situ monitoring of pea seeds germination using optical coherence tomography

**DOI:** 10.1002/pld3.428

**Published:** 2022-07-12

**Authors:** Xinhua Li, Xingyu Yang, Xiaoran Li, Zhiyi Zhao, Zijian Zhang, Hungyen Lin, Dingming Kang, Yaochun Shen

**Affiliations:** ^1^ Department of Electrical Engineering and Electronics University of Liverpool Liverpool UK; ^2^ Department of Engineering Lancaster University Lancaster UK; ^3^ College of Agronomy and Biotechnology China Agricultural University Beijing China

**Keywords:** in situ monitoring, internal structure, nondestructive, optical coherence tomography, pea seed, seed germination

## Abstract

Seed germination and uniform plant stand in the field are the most critical crop growth stages determining the final yield. Pea (*Pisum sativum L*.) seeds production is often hampered due to the seed dormancy caused by the hard seed coat. Such effect is mainly attributed to poor or uneven germination and unsynchronised seedling emergence. Understanding the time course of water intake and several critical germination indicators can reveal many features of seed germination such as rate and uniformity. This paper used optical coherence tomography (OCT), a noninvasive and cross‐sectional imaging technique, to monitor the inner structural changes throughout the germination process. A sequence of cross‐sectional OCT images of pea (*P. sativum L*.) seeds, together with additional microscopic optical images, was recorded continuously and in situ for over 40 h. OCT and microscopic images revealed the changes in the internal structure and the external shape of the pea seeds during germination, respectively. It was found that the cross‐sectional OCT images helped to identify the critical indicators distinguishing the different phases of germination pea seeds. Therefore, the presented OCT approach offers a fast and nondestructive way to precisely measure the structural indicators in different germination phases.

## 1 INTRODUCTION

Pea (*Pisum sativum L*.) seed is a grain legume of the *Fabaceae* family, widely used as a source of plant‐based protein for consumption (Chartrel et al., 2021; Henriet et al., 2021). However, most legume species in the temperate‐zone have varying degrees of physical dormancy due to their water‐impermeable seed coat. In coat‐imposed dormancy, the permeability characteristics of the seed coat may affect the timing of three phases in germination: (1) imbibition—dry seeds imbibe water and both oxygen uptake and seed mass increase, (2) lag—characterized by a metabolic plateau, and (3) completion—protrusion of the radicle through the seeds coat (Bewley et al., 2012). These three phases refer to the whole physiological process and culminate in the radicle's emergence from the enclosing seed coat. It is believed that the absorption of water by the seed (imbibition) causes the activation of metabolic processes, which ultimately leads to the expansion of the embryo and the penetration of the radicle (or other organs) through the surrounding tissues. As a critical indicator of the beginning of phase II, the emerging radicle inside the seed signalizes the dormancy is broken, and the hydration is sufficient to allow metabolic activation to take place (Nascimento & Aragão, 2004). In this case, the timing of radicle emerging can determine the ideal sow time, and the time model of water uptake can be applied to predict the water potential during the germination process. After that, the metabolic progress toward radicle emergence can be quantified (Bradford & Haigh, 1994). However, currently, the most used method to recognize the germination phases relies on the measurement of change in water content (Hardegree & Emmerich, 1992; Nonogaki et al., 2010) or the change in weight (Mehta et al., 2014).

Monitoring the features of the inner seed structure during germination can provide useful information about pea seeds that may affect germination efficiency and harvest uniformity. The ability to quantitatively image the internal structure of crop seeds is thus essential as it provides rich physical attributes for reliable and accurate analysis of crop seed quality and variety. At the same time, advances in various technologies have allowed the internal structures of these seeds to be imaged. Examples of these include scanning electron microscopy (SEM) (Coffigniez et al., 2019; Hu et al., 2009) and transmission electron microscopy (TEM) (Gallant et al., 1997; Putaux et al., 2000). Researchers have also employed hyperspectral imaging to assess the seeds (Huang et al., 2014; Mahesh et al., 2011; Zhu et al., 2011). Fourier transform near‐infrared and Raman spectroscopy has been used for the nondestructive evaluation of viable pepper seeds (Seo et al., 2016). Confocal micro‐Raman spectroscopy has also been used for the nondestructive assessment of biochemical changes related to Jamun fruit ripening (Sharma et al., 2022). However, these spectroscopic imaging methods only provide information on material composition at or near the seeds surface, and they do not provide any depth‐resolved information on the internal structures of the seeds. In order to get cross‐sectional images using these methods, the seed sample will have to be cut, and therefore, the measured seeds can no longer be used once the measurement is taken.

Optical coherence tomography (OCT) is a nondestructive imaging technology that is capable of providing cross‐sectional images of a seed with micrometer‐resolution. Apart from ophthalmic imaging application, recently, OCT has also been successfully used in characterizing pharmaceutical tablet coatings (Lin et al., 2017, 2018), soiled cotton fabric (Zhang et al., 2019) and biological tissues (Li et al., 2020). Compared with other imaging technologies that have been used for imaging seeds, OCT has a unique capability to image and resolve internal morphological variations of seed coat during seed germination, which will help to understand such a natural biological process.

Clements et al. reported the use of an OCT system to analyze the hull thickness of lupin seeds (Clements et al., 2004). Following the same research direction, Lee et al. proposed to use OCT method for screening diseases in melon (Lee et al., 2011) and cucumber seeds (Lee et al., 2012) for the first time. Recently, Yoon and Lee (Yoon & Lee, 2019) adopted the same technique to identify fungus‐infected tomato seeds. Similarly, Joshi et al. (Joshi, et al., 2021) presented the use of OCT system for subsurface imaging of rice seeds to distinguish different seed varieties. In addition, OCT techniques have also been applied for studying leaf senescence (Anna et al., 2019) and root growth in soil (Larimer et al., 2020). Three‐dimensional (3D) imaging of the internal structures of tomato seeds has also been reported by Fan and Yao (Fan & Yao, 2012).

**FIGURE 1:**
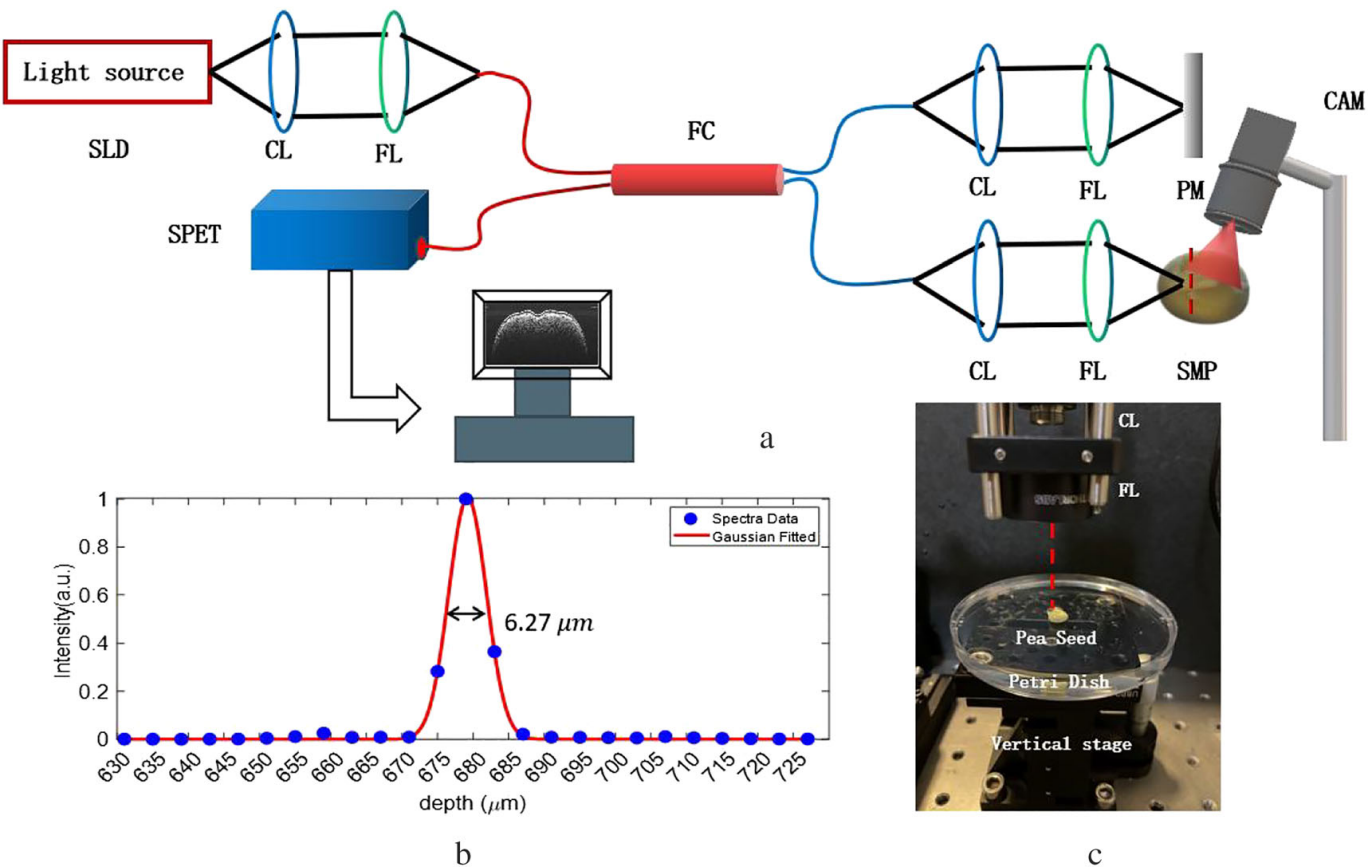
(a) The schematic diagram of the developed SD‐OCT imaging system. SLD, Superluminescent diode; CL, Collimator lens; FL, Focusing lens; FC, Fiber coupler; SPET, Spectrometer; SMP, Sample seed PM, Plane mirror. (b) The axial resolution of the OCT system; (c) A photograph of the experimental setup where the distance between the lens and the seed can be adjusted before the measurement

The application of swept‐source OCT (SS‐OCT) to monitor germination rate in chemically primed seeds was demonstrated recently (Ravichandran et al., 2017). SS‐OCT has also been used to analyze the germination rate of Capsicum annum seeds treated with growth‐promoting chemical compounds (Wijesinghe et al., 2017). A SS‐OCT system, on the one hand, allows an imaging depth of a few millimeters to be achieved. On the other hand, it usually requires the use of an expensive tunable laser as the light source (Choma et al., 2003). In contrast, the SD‐OCT system reported in this study is significantly less expensive while it provides a good axial resolution of 6.3 μm (Figure 1b) and a signal‐to‐noise ratio (SNR) sufficient for characterizing most seed samples (Li et al., 2021). Furthermore, the work reported here not only images the change of seeds internal structures but also reveals a new correlation between seed coat change and germination stages.

The metabolic activities and the internal metabolic changes in different phases during germination have been solidly proven (Bewley, 1997; Nonogaki et al., 2008). In detail: in phase I, internal pressures may cause breakage of cell walls, blistering of the cotyledon surface, and ejection of cellular contents when imbibition is excessively fast. During hydration, cellular membranes may potentially leak ions and solutes in addition to direct extrusion. In phase II, the repair of accumulated DNA damage and the rebuilding of the cellular cytoskeleton are completed, and as the young seedling establishes itself in phase III, more water is taken in using the primary accumulated stores.

However, to date, detailed investigation regarding the morphological changes during imbibition and interim phases has not been reported with any seed cultivars. The technique proposed may provide an indirect method for understanding the internal metabolic process since it demonstrated the correlation between germination phases and internal structure changes.

In this work, we proposed a high‐resolution custom‐built SD‐OCT working at 850 nm to monitor and characterize the whole seed germination process. A web camera has also been integrated into the SD‐OCT system. Both the external morphological changes and the internal structural variations of the seed samples could be recorded simultaneously. This is indispensable for a deep understanding of seed germination and for a comprehensive demonstration of the physical dormancy broken by soaking the seed. In this work, we also provide morphological indicators during imbibition and interim phases of pea seed germination. Moreover, by applying the proposed system with a specific priming technique, seeds can be picked up accurately at the end of physical dormancy broken to enable optimal priming over the traditional fresh weight method.

## 2 MATERIALS AND METHODS

### 2.1 System configuration

Figure 1 shows the schematic diagram of the SD‐OCT system used for in situ and continuous imaging of Pisum sativum seeds. In brief, the light source used is a superluminescent light‐emitting diode (SLED), providing a broadband illumination centred at 840 nm with a half width at half maximum (FWHM) bandwidth of 50 nm. The light was collimated and then coupled into the input port of a 2 × 2 fiber coupler where the light beam was split (50:50) into a reference and a sample probe beam. The reference beam was focused onto a reference mirror using an adjustable collimator (CFC‐2X‐B, Thorlabs), and the probe beam was focused onto the sample by using another identical adjustable collimator and a focusing lens. The light backscattered from the sample and the light reflected from the reference mirror were collected and subsequently combined at the fiber coupler. The resulting interferogram was measured using a high‐resolution spectrometer (Cobra SRC, Wasatch Photonics). The spectral resolution of the spectrometer is 0.09 nm, providing an imaging depth of 2.2 mm. The data acquisition rate of the spectrometer was 10,000 A‐scans/s. The SD‐OCT system has an axial resolution of 6.3 μm in pea seeds and a lateral resolution of 25 μm. The SNR was measured to be over 85 dB.

The fiber head of the sample arm was settled on a motorized translation stage (MT S25‐Z8, Thorlabs) controlled by a servo motor controller (TDC001). The scanning length across the seed was set at 6 mm during the measurement, and the seed was automatically measured every 10 min for 40 h. Each B‐scan image has 1,024 × 1,000 pixels, covering an effective imaging area of 6 mm (wide) × 2.2 mm (deep). The optical camera (BFLY‐U3‐13S2C‐CS) was mounted next to the OCT probe head. Using a camera lens with a fixed focal length of 4.5 mm could photograph the measured seed with a resolution of 644 × 482 pixels, corresponding to a functional imaging area of 11 mm × 11 mm for one seed. Before the measurement, the distance of the lens from the seed and the aperture of the camera were adjusted to ensure that the seed is in focus. The camera was then fixed aside from the measured seed samples, and the depth of focus of the camera was adjusted to be approximately 18 mm, which can accommodate any position change caused by the seed growth (the pea seed diameter change is within 3–5 mm). The lateral resolution of the microscope image was measured to be 40 μm.

### 2.2 Boundary recognition algorithm

**FIGURE 2:**
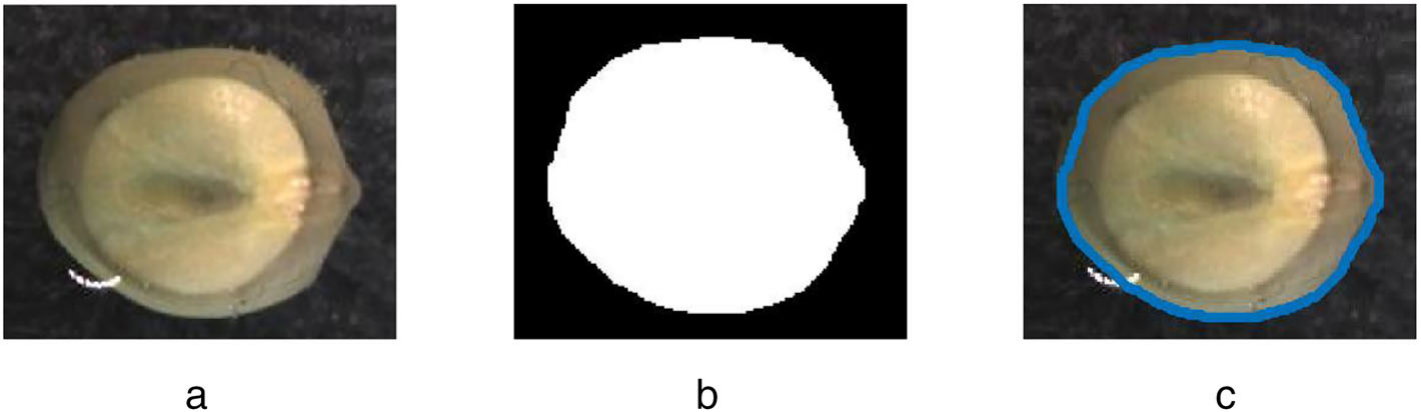
The boundary recognition algorithm. (a) The original seed images taken by the camera. (b) Is the image after the opening operation of canny edge algorithm. (c) Boundary recognition results marked by blue line on the original image, the second boundary with shadow in the middle is the water surface

In order to assess the external morphological changes of the seed samples during germination, a classic graph‐based image segmentation method was applied to the photos recorded with the web camera (Figure 2a). Step 1: binarization was applied to images with a thresholds range of 62 to 255, 45 to 255, and 30 to 255 for each of three channels (RGB color space), respectively. Step 2: the segmentation mask was created and returned in binarization image. Step 3, a Canny edge algorithm with two basic operators combined was applied to localize the boundary signals as shown in Figure 2b. Step 4, the recognized boundary was marked on the original image shown as Figure 2c. The MATLAB 2020b in this experiment was used for edge detection.

In our experiment, 10 seeds from the same batch with similar sizes were placed evenly in a clean petri dish. Again, 9 ml of distilled water (DW) is added to the Petri dish, and the hilum part was kept in the water. The weight gain of each seed was measured by a weighing scale (EMB 200‐2) every 30 min during the first 10 h, and every 5 h in the next 30 h.

### 2.3 Preparation of pea seed samples

**FIGURE 3:**
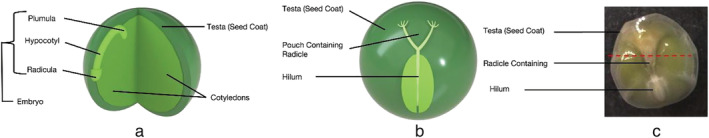
The structure of mature *Pisum sativum* seed. (a) Cross‐section for internal structure; (b) exterior structure; (c) a photo of the seed sample taken by an optical camera

All pea seeds were obtained from Sow Seed Ltd. Figure 3 shows the schematic structure of mature pea seeds. Figure 3a,b shows the cross‐section for internal structure and exterior structure of pea seeds, respectively. The pea seed was placed on a culture dish with a diameter of 15 cm, and the 9 ml DW was added to the Petri dish to ensure that the hilum part was kept in the water. All measurements were carried out at room temperature of 25 ± 2°C. The humidity was kept at 40%–45%, and the illumination conditions were also kept constant. In the subsequent germination monitoring studies, OCT images were continuously taken at the chosen place highlighted as a red dotted line (Figure 3c) which includes the plumula region, pouch region, and cotyledon with testa covering.

In order to validate the seeds' OCT images, microscope images were also taken on the cut sections of the seed. The cross‐sectional images of seed sample were first measured by OCT, and the measured seed sample was then cut in half for microscope imaging. Thus, there is no biological changes in seeds. Three pea seeds with approximately the same length (6.3 ± 0.3 mm), width (5.4 ± 0.3 mm), and height (4.7 ± 0.2 mm) were first measured to identify the best positions for monitoring the subsequent germination process. Firstly, OCT cross‐section images were taken at the areas of plumula, testa, and hilum. The samples were subsequently cut at the same area where the OCT image was taken: the first one at the plumula, the second one at the testa, and the final one at the hilum. Finally, microscope images of the cross‐section of these cut samples were taken with a 10X binocular stereo microscope with LED light (S41‐20, SWIFT).

## 3 RESULTS

### 3.1 OCT image of pea seeds and its validation by microscope

**FIGURE 4:**
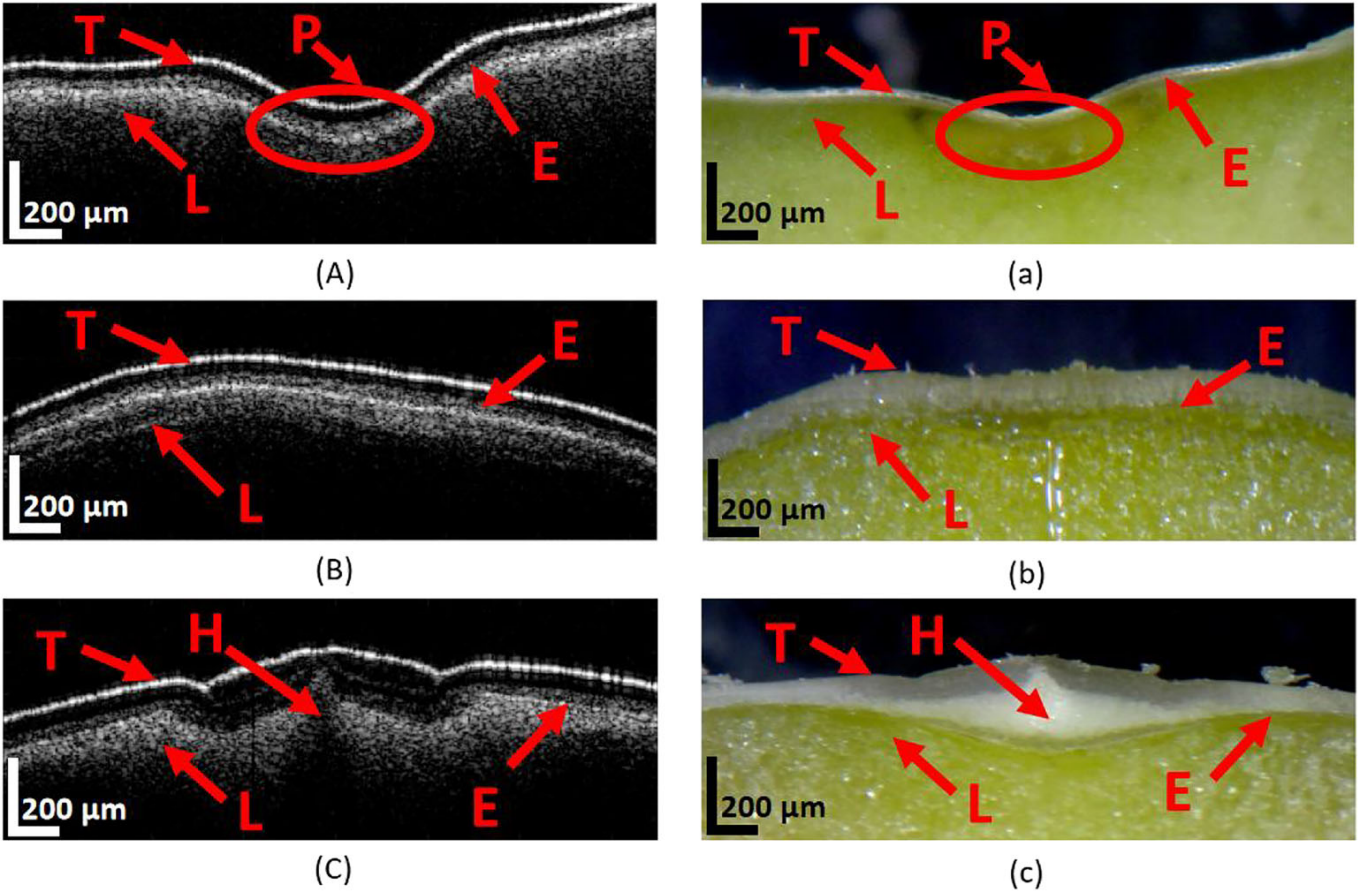
OCT image shows the cross‐sectional view of the plumula (A), testa (B), and hilum (C) regions of pea seed. Microscope image of the cut‐section of the same region of pea seeds showing the corresponding plumula (a), testa (b), and hilum (c) regions. T: Testa; P: Pouch containing; L: cotyledon; H: Hilum; E: Endocarp

Before studying seed germination with the SD‐OCT, we validated its cross‐sectional imaging by measuring a dry pea seed and comparing the results to those imaged by a commercial microscope. Three regions of interest of the seed sample were firstly imaged by the SD‐OCT nondestructively, as shown in Figure 4A–C. As a result, the seed tissues like the plumula, testa, endocarp, pouch, and cotyledon can be observed. The measured seed sample was then cut in half for microscope imaging. Nearly the same tissue structures were found in the microscope photos after magnifying the seed about 10 times, as represented in Figure 4a–c, which validated the SD‐OCT. Figure 4 illustrates the cross‐sectional OCT and microscope images of the pea seed samples. Figure 4A and a shows the cross‐sectional view of the plumula region; the testa, endocarp, pouch, cotyledon can all be observed. Figure 4B and b shows the cross‐sectional images of the testa, endocarp, and cotyledon. In Figure 4C and c displays the view of the hilum region with the testa, endocarp, hilum, and cotyledon clearly outlined. The OCT images are in general agreement with the optical microscope images.

We have chosen the pouch containing part (Figure 4A), where the radicle inside can be first identified, was chosen as the monitoring site in the following continuous measurement of the seed germination process, aiming to determine the accurate time course of the internal structural changes in different phases.

### 3.2 Continuous monitoring of germination process by OCT

**FIGURE 5:**
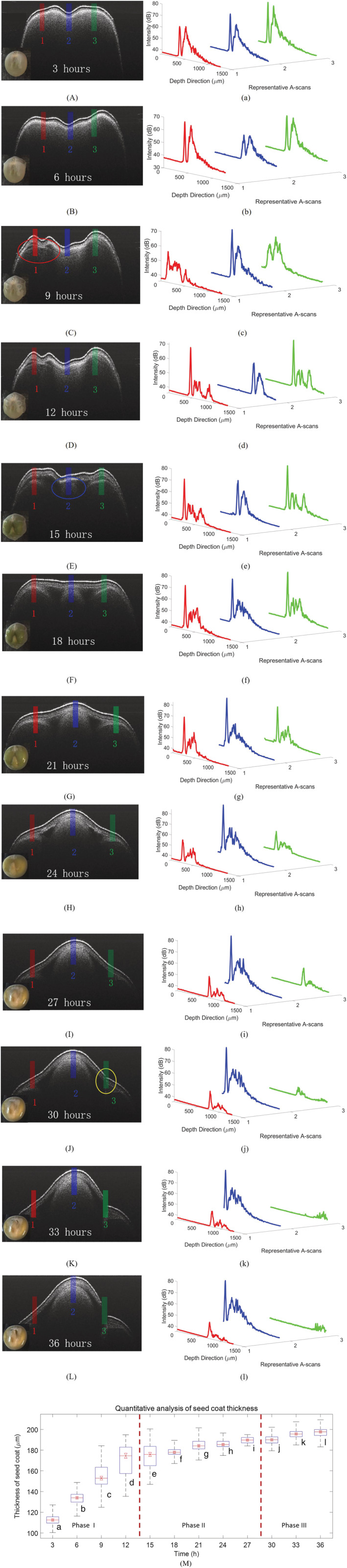
Typical OCT images chosen with an interval of 3 h, revealing different phases of the germination: Phase I (A‐D), phase II (E‐I), and III (J‐L) and their corresponding A‐scan waveforms (a‐l) taken from position1, 2, and 3, marked by red, blue, and green in B‐scans, respectively. The cotyledon layer becomes visible in (C) marked by red circle; the radicle first observed in (E) marked by blue circle; the seed coat cracked in (J) marked by yellow circle. (M) Is quantitative analysis of seed coat thickness with a boxplot for three phases. The maximum whisker length is specified as two times the interquartile difference in the box plot. The horizontal line within the box indicates the median and the bottom and top edges of the box indicate the first quartile and the third quartile, respectively. The red dotted lines in (M) are used to distinguish the three phases

To investigate the feasibility of using OCT cross‐sectional imaging to recognize the characteristic pattern indicators in different phases, we consecutively monitored seed germination and both external and internal morphological changes. We collected 240 SD‐OCT images and web camera photos. These results were obtained within 40 h along with seed germination after soaking the seed with DW. Figure 5 illustrates the representative results of seed morphological changes every 3 h over the germination period. We also plotted A‐scan waveforms next to each of their corresponding B‐scan images. It is worth pointing out that the A‐scan waveforms were selected to involve radical and cotyledon regions.

The seed coat absorbed water during the entire imbibition phase until it entered the lag phase. Figure 5A–D shows the details of this process (phase I). As shown in Figure 5A and more clearly in the enlarged view shown in Figure 4A, three layers, that is, the testa, the endocarp, and the cotyledon, are visible in the OCT image. A close look at all 18 OCT images taken in the first 3 h reveals no significant changes compared with their original state (e.g., dry seed). In the early stage of the phase I, as a large portion of air pores exist in seed coats and cotyledons, the medium's heterogeneity leads to strong scattering, resulting in relatively larger OCT signals, especially the second peak of the A‐scan waveforms in Figure 5a,b. During the imbibition phase, the different levels of water uptake by the seed coat and various internal parts of the seed lead to uneven surface tension. External morphological variations like protuberances on the seed coat can be observed from the photos taken by the web camera (the insets of Figure 5C,D). From the subsurface view, we identified that the water uptake changed internal inhomogeneity and allowed light to transmit to deeper tissues because of the replacement of air pores with water. As a result, internal variations like the formation of cotyledons and radicle regions could be detected by the SD‐OCT, as shown in Figure 5C,D. These changes can also be revealed by the reduced signal intensity of the endocarp layer and the appearance of new peaks corresponding to the two cotyledons (refer to No.1 Ascan (red) and No. 3 Ascan (green) in Figure 5d). Furthermore, we observed that the cotyledon layer became more apparent in the next few hours. An increase in the distance between the radicle and the seed coat is necessary to provide space for embryo expansion. Meanwhile, the layers of cotyledon clear emerged on both the left and right sides with the absorbency swelling of the radicle in the middle. Phase I, during which water is ingested, is mostly a physical process, and physiological processes may begin within minutes of a cell being hydrated, even before all of the seed tissues have been completely imbibed. The morphological and anatomic changes found in space and time characterized the imbibition step, indicating that dormancy was broken once water was ingested.

In the lag phase, the water content of the seed is moderately constant. Figure 5E–I illustrates it (phase II). In this phase, the seed begins to metabolize the available store of carbohydrates to make proteins. Consequently, tender burgeons were observed in the OCT image for the first time, as shown in Figure 5E. Figure 5E illustrates the start of the lag phase, evident from the continuous growth of the radicle marked by the blue circle (the central portion of Figure 5E–I). The intensity of the backscattered light becomes relatively strong in the middle, represented as the second peak in the No. 2 A‐scan (blue), as illustrated in Figure 5e. With the emergence of radicle inside the seed, the up surface of the radicle was getting merged with seed coat; it may cause the refractive index (RI) of this component to have a significant difference from its surroundings. The subsequent B‐scan images show that it is the location of the radicle emerging. It can be seen Figure 5e that the second peak is obvious but does not exceed the first peak in No. 2 A‐scan (blue), suggesting that the stronger backscattered light is most likely caused by the change in RI due to the emerging radicle inside the seed sample. It is noted that both the position and the shape of the seed surface/interface will change with the growth of the radicle. The relative position of the surface/interface of the seed is well within the depth of focus of the OCT probe; thus, the change of surface/interface position will not cause significant change of the relative intensity of the collected backscattered light while the change of surface/interface shape may do. Phase II is also the appropriate time to end the hydropriming and sow the seed. With the further imbibition and radicle growth inside, the radicle's heterogeneity also increased, leading to a surge in scattering, as shown in No. 2 A‐scan (blue) in Figure 5g. It is possible to comparethese results with No. 2 A‐scan (blue) in Figure 5e,f for example. With the nascent radicle growing and swelling sustainably, the seed size continues to increase from the 15th hour. The surface of the seed coat deforms due to the expansion of the radicle inside as shown in the inserted figure in Figure 5H,I. During phase II, the water level of the seeds remains relatively constant, while metabolic activities rise as a result of the extensive transcription of new genes. The emergence of the radicle through the surrounding structures at the conclusion of this phase means the completion of dormancy breaking.

Phase III sees an increase in water intake as the young seedling establishes itself. Figure 5J–L corresponds to the final phase (phase III), which is the last step of germination. One of the main characteristics of this phase is the radicle going out of the seed coat. The radicle completely broke through the seed coat and became visible to human eyes, as shown in the inserted figure in Figure 5J–L. The lower part of the radicle subsequently becomes roots, which are the indicator of a well‐germinated seed. Figure 5J also shows that the expanded radicle region finally resulted in the cracking of the seed coat marked. The yellow circle represents the end of the lag phase. Furthermore, the fracture grows inthe last phase of the germination, as shown in Figure 5J–K. Phase III involves increased water absorption as the immature plant grows and gets established, allowing the seedling to make use of the main accumulated reserves. In Figure 5M, the maximum whisker length is specified as two times the interquartile difference in the box plot. On each box, a horizontal line within the box indicates the median of the seed coat, and the bottom and top edges of the box indicate the first quartile and the third quartile, respectively. The whiskers extend to the most extreme data points. The spacings in each subsection of the box plot indicate the degree of spread of the seed coat. It should be noted that the lengths of boxes in 9, 12, and 15 are significantly longer than the others due to the uneven seed coat surface during this time. The average and the standard deviation in thickness quantifications are listed in Table 1:

**TABLE 1:** The thickness quantifications of seed coats measured in different times

Time (h)	Average thickness (um)	Standard deviation (μm)	Time (h)	Average thickness (um)	Standard deviation (μm)
3	112	4.6	21	185	5.8
6	133	5.4	24	186	4.1
9	155	13.1	27	190	2.9
12	170	15.3	30	190	4.3
15	175	12.6	33	196	4.0
18	178	4.2	36	197	4.8

**FIGURE 6:**
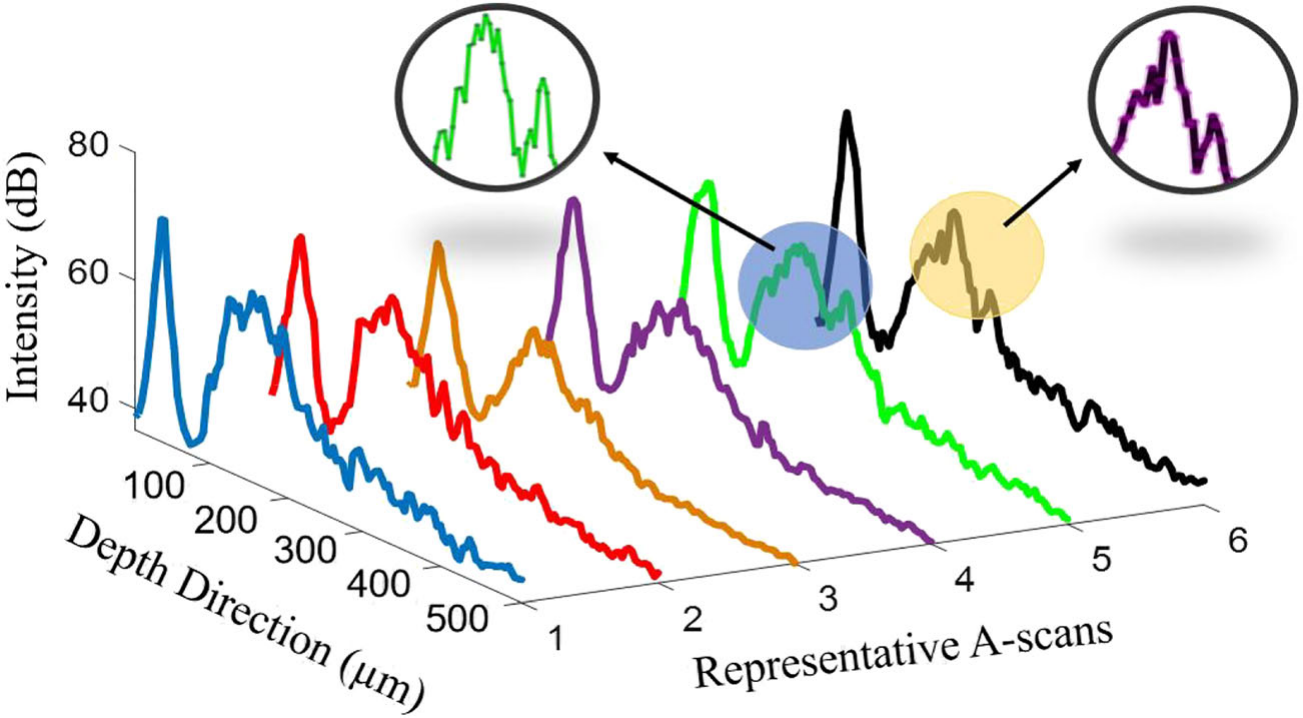
The 20 averaged depth profiles from 13th to 14th hour with time interval of 10 min. In the 5th A‐scan (acquired in 13th hour 40th min), a newly emerged peak in depth profile accompanied by the second peak marked by the arrows, indicating the radicle in subsurface can be distinguished for the first time and it is also the indicator for the beginning of the lag phase

In order to precisely pinpoint the start of the second phases of the whole priming process, 20 A‐scan waveforms were averaged and six averaged depth profiles (A‐scans) between the 13th to 14th hour with a time interval of 10 min were calculated (refer to Figure 6). In the first 4 A‐scans, there were two prominent peaks in each profile. This indicates that the pea seed is still in the first phase, and the radicle inside is still invisible. In the 5th A‐scan (at 13 h and 40 min) and 6th A‐scan (at 13 h and 50 min), a newly emerged peak accompanied by the second peak (marked with a circle) becomes visible. It is a significant indicator for the beginning of the lag phase, and the radicle in the subsurface can be distinguished for the first time. Thus, a newly emerged radicle in the subsurface indicates that the seeds were released from dormancy. In this case, phase II is a proper timing for sowing, making sure the seeds can complete germination at roughly the same time in the following phase III. We believe that these observable signatures of OCT signal could be used as indicators to discriminate the sowing time.

### 3.3 Quantitative analysis with the change of seed coat thickness, seed size and seed weight (golden standard) in seed priming

For pea seeds, physical dormancy is a common effect due to the water permeability of the seed coat. A thick and hard seed coat may delay embryo development and radicle growth. Thus, the seed coat thickness will have an impact on seed germination rate and speed. Here, we calculate the coating thickness from the recorded B‐scan images.

**FIGURE 7:**
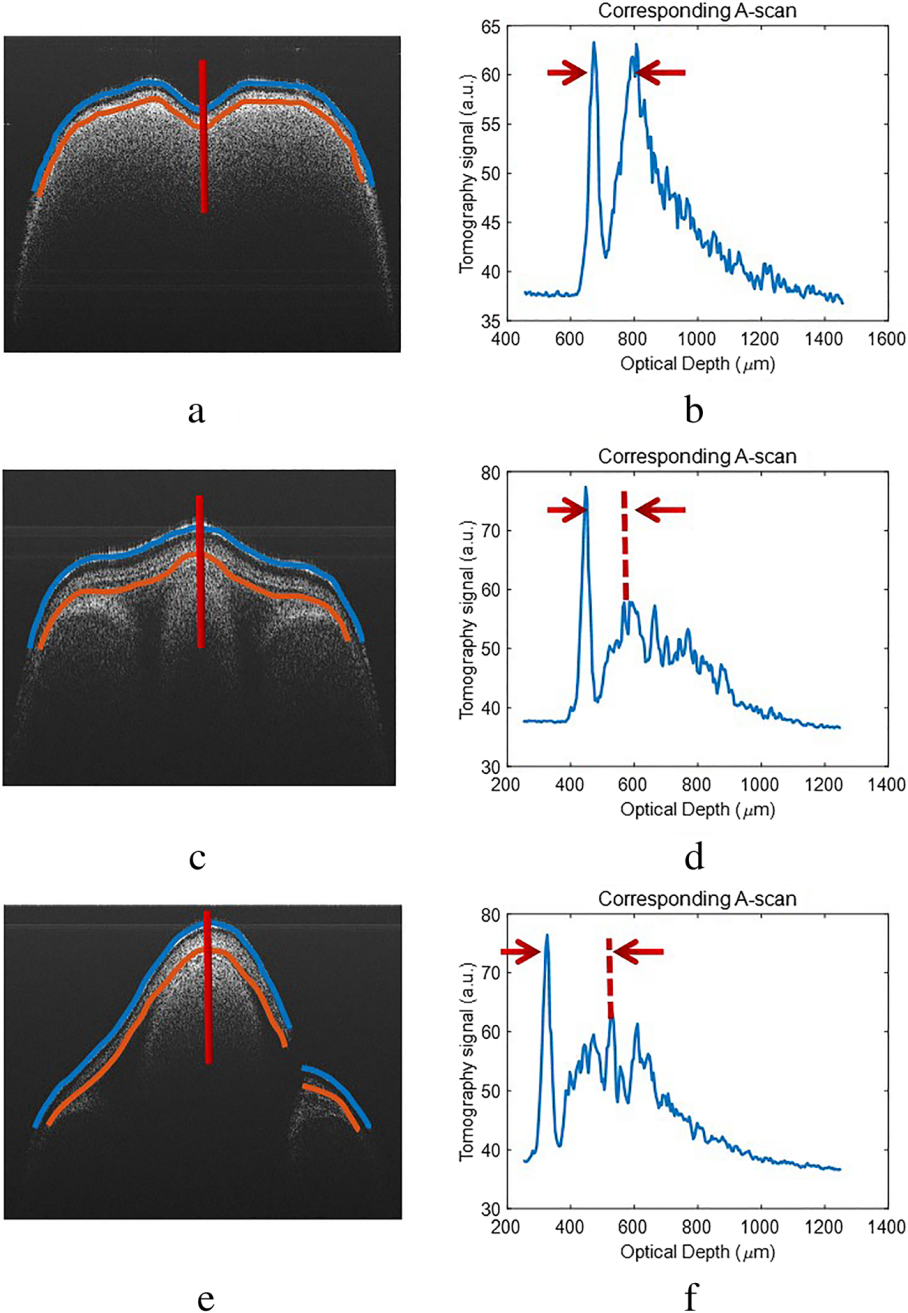
Three typical B‐scan OCT images of a pea seed obtained using SD‐OCT showing three different phases: Phase I (a); phase II (c), and phase III (e). Blue lines and orange lines in (a), (c), and (e) are to indicate the upper and lower interfaces of the seed coat, respectively. The red lines mark the central transverse position where 20 A‐scan waveforms are taken to provide the averaged the averaged waveform shown in (b), (d), and (f), respectively. The distance between two red arrows indicates the thickness of the seed coat at the central position

The A‐scan waveforms in Figure 7b,d,f were acquired by averaging 20 spectra centered around the red line marked on the Figure 7a. The distance between two red arrows in the averaged A‐scan waveform provides a quick estimation of the thickness of the seed coat at the central part of B‐scans.

**FIGURE 8:**
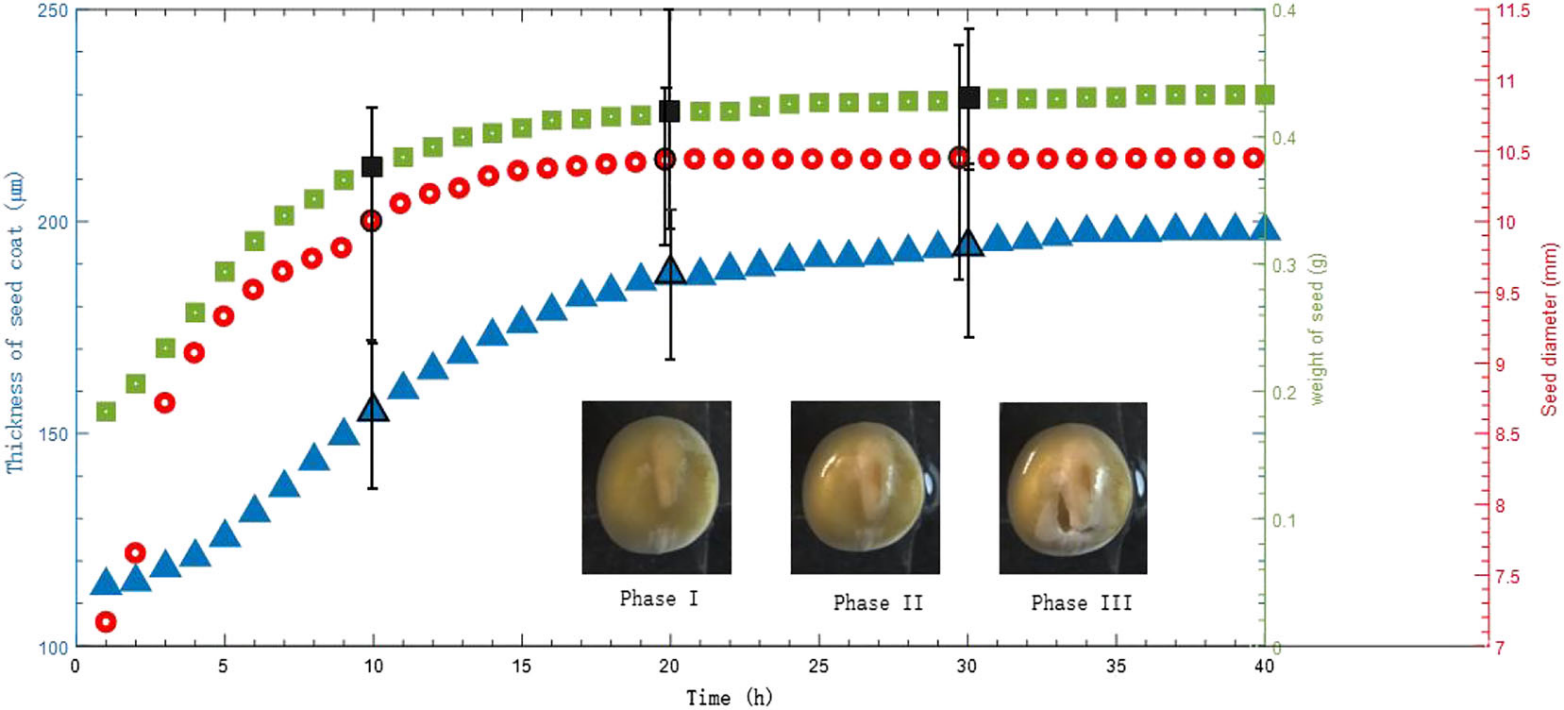
Time course analysis of weight of seed (golden standard), thickness of seed coat and seed diameter in 40 hours after seed soaked in water. Ten seeds are monitored in this measurement and averaged seed coat thickness change is marked as the blue triangle, averaged weight change marked by the green square and the diameter change marked by the red dot

To calculate the thickness more precisely and reliably, 60 points (equally spaced in the lateral direction) were manually marked on the surface and the interface of the seed samples, as shown in Figure 7a,c,e. These points were then used to produce the best‐fitted curves of the surface and the interface of the seed sample by using Piecewise Cubic Hermite Interpolating Polynomial (PCHIP). The resultant best fitted curves were marked as blue and orange curves in Figure 7a,c,e. The thickness of the seed coat can subsequently be calculated as the distance between these two best‐fitted curves. Figure 5 shows the quantitative analysis of the calculated thickness. The RI of seed coat used is 1.42 (Ravichandran et al., 2017). Figure 8 shows the coating thickness as a function of time. As a comparison, Figure 8 also shows the seed diameters calculated from the optical images.

Figure 8 shows the measured time‐dependent weight, size (measured from microscope images) and seed coat thickness (measured from OCT images). The correlation analysis was carried out according to Equation 1), where *x* represents the averaged weight as the golden standard, *y* represent the seed coat thickness or the size of seed, and *r* is their correlation indexes with the weight, respectively. The correlation coefficient of the weight and thickness of seed coat is 0.951, while the correlation index of the weight change and diameter change is 0.972. These rates indicate that both the seed coat thickness and diameter have a significant correlation (|*r*| > 0.95) with seed weight, representing the golden standard in germination.

(1) $$r=\frac{\sum \left(x_{i}-\bar{x}\right)\left(y_{i}-\bar{y}\right)}{\sqrt{\sum {\left(x_{i}-\bar{x}\right)}^{2}}\sum {\left(y_{i}-\bar{y}\right)}^{2}}$$

It is evident that the results of the proposed OCT method agree with that of the weight measurement method, which is the current golden standard for determining three phases. Furthermore, OCT can provide critical structure indicators for precisely determining different phases precisely in a noninvasive and noncontact way.

Gravimetric method is a classic method used for determining the germination status of seeds. However, Gravimetric method requires the seeds to be taken away from the germination environment for measurement, and this will change the growth environment of the seeds and interrupts the seed germination process. In addition, to obtain accurate results, the gravimetric method normally requires the measurement of many seeds to obtain averaged weight gain, and this is time‐consuming (Garnczarska et al., 2007).

OCT as a nondestructive imaging technology that can determine the germination phases without the need to interrupt the seed germination process. OCT images can detect changes of internal structures of seed sample including coat thickness. The detected changes correlate with the seed weight information provided by the golden standard method. It can be concluded that the OCT can provide structure indicators for determining different phases in a noninvasive way. In contrast, the gravimetric method can only provide the seed weight information rather than the internal structure information of seeds (Manz et al., 2005), and the gravimetric method will cause interruption to the germination process.

**FIGURE 9:**
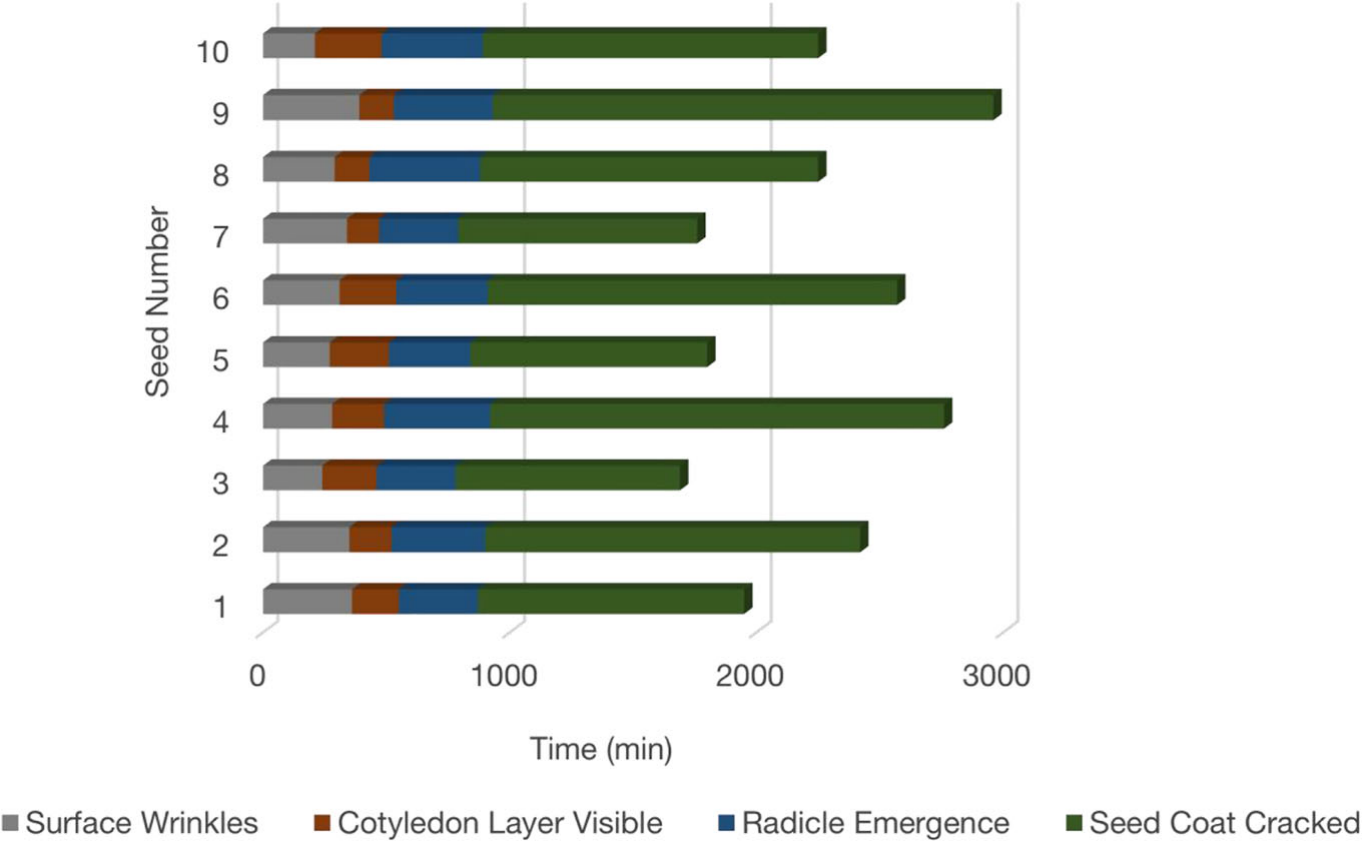
The timing of the emergence of critical indicators during the germination of 10 seeds. First indicator (the surface wrinkles) is marked by gray bar; second indicator (cotyledon layer visible) is marked by brown bar; the third indicator (radicle emergence) is marked by blue bar, and fourth indicator (seed coat cracked) is marked by green bar

To quantitativly analyze the critical stages of the seed germination process, Figure 9 summarizes the timing of critical indicators of 10 pea seeds from the same batch. These four key indicators, including surface wrinkle, cotyledon layer, radicle emergence and seed coat cracked, outline clear boundaries of each stage during seed germination. It is noteworthy that OCT could resolve all four indicators, while naked eyes can only identify two of these four indicators. It was also found that the timing of seed coat cracking looks more relevant to the timing of the radicle emerging, while it is less relevant to the time of surface wrinkling and radicle emergence. We then conclude that the OCT results can be used in germination prediction in future work.

## 4 CONCLUSION

In this study, we have demonstrated the capability of SD‐OCT with an integrated camera for the in situ and nondestructive evaluation of different phases of seed germination. OCT cross‐sectional iamge and the optical imaging provide complementary information regarding the seeds' internal structure and external shape throughout the gemination process. In addition, the proposed nondestructive method can provide time course‐related data to resolve the irregular sprouting issue that emerged during harvesting and improve germination efficiency. Particularly, our fiber‐based OCT system is compact and can be packaged as a portable instrument, which suits the actual needs of the agricultural industry. This method can help to have better understanding of detailed internal structure change during germination, and it can also be applied to study other optimal priming methods using chemical solutions to determine the proper timing for seed sowing to ensure better crop yield.

Since the seed coat, endosperm, and embryo morphological structures are closely related to seed viability and environment characteristics, the reported OCT technology can be further extended to nondestructive detection of endosperm and embryo structure at the presowing screening stage. The OCT can be used for in situ rapid identification and screening of high‐quality seed in the future.

## AUTHOR CONTRIBUTIONS

Yaochun Shen conceived and supervised the study; Xinhua Li designed the study, done the experiment, and wrote the article; Xingyu Yang and Xiaoran Li contributed in the development of the coding and the optical system adjustment; Zhiyi Zhao was involved in the system debugging and light source integration; Zijian Zhang was involved in the SD‐OCT system development; Dingming Kang supervised the plant part and helped with writing the original draft of the manuscript; the dimension change and analysis under the supervision of Hungyen Lin. All of the authors have read and approved the final version of the manuscript with further critical input.

## CONFLICT OF INTEREST

The Authors did not report any conflict of interest.

## References

[pld3428-bib-0001] Anna, T. , Chakraborty, S. , Cheng, C.‐Y. , Srivastava, V. , Chiou, A. , & W‐CJSr, K. (2019). Elucidation of microstructural changes in leaves during senescence using spectral domain optical coherence tomography. Scientific Reports, 9, 1–10. 10.1038/s41598-018-38165-3 30718740 PMC6362184

[pld3428-bib-0039] Bewley, J. D. (1997). Seed germination and dormancy. The Plant Cell, 1055–1066. 10.1105/tpc.9.7.1055 12237375 PMC156979

[pld3428-bib-0002] Bewley, J. D. , Bradford, K. , & Hilhorst, H. (2012). Seeds: Physiology of development, germination and dormancy. Springer Science & Business Media. 10.1007/978-1-4614-4693-4_4

[pld3428-bib-0003] Bradford, K. J. , & Haigh, A. M. (1994). Relationship between accumulated hydrothermal time during seed priming and subsequent seed germination rates. Seed Science Research, 4(2), 63–69. 10.1017/S0960258500002038

[pld3428-bib-0004] Chartrel, V. , Dugat‐Bony, E. , Sarthou, A.‐S. , Huchette, S. , Bonnarme, P. , & Irlinger, F. (2021). The microbial community associated with pea seeds (Pisum sativum) of different geographical origins. Plant and Soil, 462, 1–23. 10.1007/s11104-021-04856-6

[pld3428-bib-0038] Choma, M. , Sarunic, M. , Yang, C. , & Izatt, J. (2003). Sensitivity advantage of swept source and fourier domain optical coherence tomography. Optics Express, 11(18), 2183–2189. 10.1364/oe.11.002183 19466106

[pld3428-bib-0005] Clements, J. , Zvyagin, A. , Silva, K. , Wanner, T. , Sampson, D. , & Cowling, W. (2004). Optical coherence tomography as a novel tool for non‐destructive measurement of the hull thickness of lupin seeds. Plant Breeding, 123(3), 266–270. 10.1111/j.1439-0523.2004.00989.x

[pld3428-bib-0006] Coffigniez, F. , Briffaz, A. , Mestres, C. , Akissoé, L. , Bohuon, P. , & El Maâtaoui, M. J. F. R. I. (2019). Impact of soaking process on the microstructure of cowpea seeds in relation to solid losses and water absorption. Food Research International, 119, 268–275. 10.1016/j.foodres.2019.02.010 30884656

[pld3428-bib-0007] Fan, C. , & Yao, G. (2012). 3D imaging of tomato seeds using frequency domain optical coherence tomography. Sensing for Agriculture and Food Quality and Safety IV, 8369: SPIE, 101–106.

[pld3428-bib-0008] Gallant, D. J. , Bouchet, B. , & PMJCp, B. (1997). Microscopy of starch: evidence of a new level of granule organization. Carbohydrate Polymers, 32(3–4), 177–191. 10.1016/S0144-8617(97)00008-8

[pld3428-bib-0009] Garnczarska, M. , Zalewski, T. , & Kempka, M. J. P. P. (2007). Water uptake and distribution in germinating lupine seeds studied by magnetic resonance imaging and NMR spectroscopy. Physiologia Plantarum, 130, 23–32. 10.1111/j.1399-3054.2007.00883.x

[pld3428-bib-0010] Hardegree, S. P. , & Emmerich, W. E. J. J. E. B. (1992). Effect of matric‐priming duration and priming water potential on germination of four grasses. Journal of Experimental Botany, 43, 233–238. 10.1093/jxb/43.2.233

[pld3428-bib-0011] Henriet, C. , Balliau, T. , Aimé, D. , Le Signor, C. , Kreplak, J. , Zivy, M. , Gallardo, K. , & Vernoud, V. (2021). Proteomics of developing pea seeds reveals a complex antioxidant network underlying the response to sulfur deficiency and water stress. Journal of Experimental Botany., 72, 2611–2626. 10.1093/jxb/eraa571 33558872

[pld3428-bib-0012] Hu, F. , Zhang, G.‐N. , & J‐JJM, W. (2009). Scanning electron microscopy studies of antennal sensilla of bruchid beetles, *Callosobruchus chinensis* (L.) and *Callosobruchus maculatus* (F.)(Coleoptera: Bruchidae). Micron, 40, 320–326. 10.1016/j.micron.2008.11.001 19101159

[pld3428-bib-0013] Huang, M. , Wang, Q. , Zhang, M. , & QJJoFE, Z. (2014). Prediction of color and moisture content for vegetable soybean during drying using hyperspectral imaging technology. Journal of Food Engineering, 128, 24–30. 10.1016/j.jfoodeng.2013.12.008

[pld3428-bib-0015] Joshi, D. , Butola, A. , Kanade, S. R. , Prasad, D. K. , Mithra, S. A. , Singh, N. , Bisht, D. S. , Mehta, D. S. J. O. , & Technology, L. (2021). Label‐free non‐invasive classification of rice seeds using optical coherence tomography assisted with deep neural network. Optics & Laser Technology, 137, 106861. 10.1016/j.optlastec.2020.106861

[pld3428-bib-0016] Larimer, C. J. , Denis, E. H. , Suter, J. D. , & Moran, J. J. J. A. O. (2020). Optical coherence tomography imaging of plant root growth in soil, Applied Optics., 59(8), 2474–2481. 10.1364/AO.384674 32225791

[pld3428-bib-0017] Lee, C. , Lee, S.‐Y. , Kim, J.‐Y. , Jung, H.‐Y. , & Kim, J. (2011). Optical sensing method for screening disease in melon seeds by using optical coherence tomography. Sensors, 11(10), 9467–9477. 10.3390/s111009467 22163706 PMC3231267

[pld3428-bib-0018] Lee, S.‐Y. , Lee, C. , Kim, J. , & Jung, H.‐Y. (2012). Application of optical coherence tomography to detect cucumber green mottle mosaic virus (CGMMV) infected cucumber seed. Horticulture, Environment, and Biotechnology, 53, 428–433. 10.1007/s13580-012-0071-x

[pld3428-bib-0019] Li, X. , Lawman, S. , Williams, B. M. , Ye, S. , Shen, Y. , & Zheng, Y. (2020). Simultaneous optical coherence tomography and Scheimpflug imaging using the same incident light. Optics Express, 28(26), 39660–39676. 10.1364/OE.405643 33379511

[pld3428-bib-0020] Li, X. , Yang, X. , Zhang, Z. , Kang, D. , & Shen, Y. (2021). Creating seed coat catalog using spectral domain optical coherence tomography. In 2021 International Conference of Optical Imaging and Measurement (ICOIM) (pp. 14–19). IEEE.

[pld3428-bib-0021] Lin, H. , Dong, Y. , Markl, D. , Zhang, Z. , Shen, Y. , & Zeitler, J. A. (2017). Pharmaceutical film coating catalog for spectral domain optical coherence tomography. Journal of Pharmaceutical Sciences, 106, 3171–3176. 10.1016/j.xphs.2017.05.032 28624417

[pld3428-bib-0022] Lin, H. , Zhang, Z. , Markl, D. , Zeitler, J. A. , & Shen, Y. (2018). A review of the applications of OCT for analysing pharmaceutical film coatings. Applied Sciences, 8(12), 2700–2711. 10.3390/app8122700

[pld3428-bib-0023] Mahesh, S. , Jayas, D. S. , Paliwal, J. , White, N. D. 2011. Near‐infrared hyperspectral imaging for protein and hardness predictions of bulk samples of western canadian wheat from different locations and crop years using multivariate regression models. CSBE/SCGAB annual conference *(*Winnipeg, Manitoba).

[pld3428-bib-0024] Manz, B. , Muller, K. , Kucera, B. , Volke, F. , & GJPp, L.‐M. (2005). Water uptake and distribution in germinating tobacco seeds investigated in vivo by nuclear magnetic resonance imaging. Plant Physiology, 138(3), 1538–1551. 10.1104/pp.105.061663 15980194 PMC1176424

[pld3428-bib-0025] Mehta, D. , Kanwar, H. , Thakur, A. , Thakur, S. , KJIJoB‐r, T. , & Management, S. (2014). Standardization of seed hydro‐priming duration in bitter gourd, *Momordica charantia* L. International Journal of Bio‐resource and Stress Management, 5, 98–101. 10.5958/j.0976-4038.5.1.019

[pld3428-bib-0026] Nascimento, W. M. , & Aragão, F. A. S. (2004). Muskmelon seed priming in relation to seed vigor. Scientia Agricola, 61, 114–117. 10.1590/S0103-90162004000100019

[pld3428-bib-0027] Nonogaki, H. , Bassel, G. W. , & Bewley, J. D. J. P. S. (2010). Germination—still a mystery. Plant Science, 179(6), 574–581. 10.1016/j.plantsci.2010.02.010

[pld3428-bib-0028] Nonogaki, H. , Chen, F. , & KJJAPr, B. (2008). 11 Mechanisms and genes involved in germination sensu stricto. Seed Development, Dormancy, Germination, 27, 264–304. 10.1002/9780470988848.ch11

[pld3428-bib-0029] Putaux, J.‐L. , Buleon, A. , & Chanzy, H. J. M. (2000). Network formation in dilute amylose and amylopectin studied by TEM. Macromolecules, 33(17), 6416–6422. 10.1021/ma000242j

[pld3428-bib-0030] Ravichandran, N. K. , Wijesinghe, R. E. , Shirazi, M. F. , Kim, J. , Jung, H.‐Y. , Jeon, M. , & Lee, S.‐Y. (2017). In vivo non‐destructive monitoring of capsicum annuum seed growth with diverse nacl concentrations using optical detection technique. Sensors, 17(12), 2887–2898. 10.3390/s17122887 PMC5751711 29231871

[pld3428-bib-0031] Seo, Y.‐W. , Ahn, C. K. , Lee, H. , Park, E. , Mo, C. , & B‐KJJoBE, C. (2016). Non‐destructive sorting techniques for viable pepper (*Capsicum annuum* L.) seeds using Fourier transform near‐infrared and raman spectroscopy. Journal of Biosystems Engineering, 41(1), 51–59. 10.5307/JBE.2016.41.1.051

[pld3428-bib-0032] Sharma, S. , Bharti, A. , Singh, R. , & Uttam, K. J. A. L. (2022). Non‐Destructive, Label Free Evaluation of the Biochemical Profile Associated With the Growth and Ripening Process of Jamun Fruit by Confocal Micro Raman Spectroscopy. Analytical Letters, 55, 812–827. 10.1080/00032719.2021.1967968

[pld3428-bib-0037] Wijesinghe, R. E. , Lee, S.‐Y. , Kim, P. , Jung, H.‐Y. , Jeon, M. , & Kim, J. (2017). Optical sensing method to analyze germination rate of Capsicum annum seeds treated with growth‐promoting chemical compounds using optical coherence tomography. Journal of Biomedical Optics, 22(9), 091502‐1‐091502‐7. 10.1117/1.jbo.22.9.091502 28055058

[pld3428-bib-0033] Yoon, T. , & Lee, B. H. (2019). Identification of fungus‐infected tomato seeds based on full‐field optical coherence tomography. Current Optics and Photonics, 3, 571–576.

[pld3428-bib-0034] Zhang, Z. , Ikpatt, U. , Lawman, S. , Williams, B. , Zheng, Y. , Lin, H. , & Shen, Y. (2019). Sub‐surface imaging of soiled cotton fabric using full‐field optical coherence tomography. Optics Express, 27(10), 13951–13964. 10.1364/OE.27.013951 31163852

[pld3428-bib-0035] Zhu, D. , Wang, K. , Zhang, D. , Huang, W. , Yang, G. , Ma, Z. , & Wang, C. J. S. L. (2011). Quality assessment of crop seeds by near‐infrared hyperspectral imaging. Sensor Letters, 9(3), 1144–1150. 10.1166/sl.2011.1377

